# Genome-wide association study using haplotype alleles for the evaluation of reproductive traits in Nelore cattle

**DOI:** 10.1371/journal.pone.0201876

**Published:** 2018-08-08

**Authors:** André Vieira do Nascimento, Ândrea Renata da Silva Romero, Yuri Tani Utsunomiya, Adam Taiti Harth Utsunomiya, Diercles Francisco Cardoso, Haroldo Henrique Rezende Neves, Roberto Carvalheiro, José Fernando Garcia, Alexeia Barufatti Grisolia

**Affiliations:** 1 Faculdade de Ciências Biológicas e Ambientais, Universidade Federal da Grande Dourados, UFGD, Dourados, Mato Grosso do Sul, Brazil; 2 Departamento de Zootecnia, Faculdade de Ciências Agrárias e Veterinárias, UNESP, Jaboticabal, São Paulo, Brazil; 3 Departamento de Medicina Veterinária Preventiva e Reprodução Animal, Faculdade de Ciências Agrárias e Veterinárias, UNESP, Jaboticabal, São Paulo, Brazil; 4 Departamento de Apoio, Produção e Saúde Animal, Faculdade de Medicina Veterinária de Araçatuba, UNESP, Araçatuba, São Paulo, Brazil; 5 GenSys Consultores Associados S/S Ltda, Porto Alegre, Brazil; 6 International Atomic Energy Agency (IAEA), Collaborating Centre on Animal Genomics and Bioinformatics, Araçatuba, São Paulo, Brazil; INIA, SPAIN

## Abstract

Zebu cattle (*Bos taurus indicus*) are highly adapted to tropical regions. However, females reach puberty after taurine heifers, which affects the economic efficiency of beef cattle breeding in the tropical regions. The aims of this study were to establish associations between the haplotype alleles of the bovine genome and age at first calving (AFC) in the Nelore cattle, and to identify the genes and quantitative trait loci (QTL) related to this phenotype. A total of 2,273 Nelore cattle (995 males and 1,278 females) genotyped using the Illumina BovineHD BeadChip were used in the current study. The association analysis included females with valid first calving records as well as open heifers. Linkage disequilibrium (LD) analysis among the markers was performed using blocks of 5, 10, and 15 markers, which were determined by sliding windows shifting one marker at a time. Then, the haplotype block size to be used in the association study was chosen based on the highest r^2^ average among the SNPs in the block. The five HapAlleles most strongly associated with the trait (top five) were considered as significant associations. The results of the analysis revealed four genomic regions related to AFC, which overlapped with 20 QTL of the reproductive traits reported previously. Furthermore, there were 19 genes related to reproduction in those regions. In conclusion, the use of haplotypes allowed the detection of chromosomal regions associated with AFC in Nelore cattle, and provided the basis for elucidating the mechanisms underlying this trait.

## 2. Introduction

Indicine cattle (*Bos taurus indicus*) are well-adapted to tropical environments, because of attributes like heat tolerance and partial tick resistance. However, as a general rule, indicine heifers present inferior reproductive performances, regarding the onset of puberty, in comparison with taurine cattle (*Bos taurus taurus*) [1]. Such late onset of puberty negatively affects the economic efficiency of beef cattle breeding and restricts the genetic improvement of cattle [2], and is, thus, becoming a key concern in the tropical countries. In Brazil, where beef production is primarily based on indicine breeds, Nelore heifers selected for growth reach the onset of puberty at about 23 months [3]. Anticipating this stage would increase the producer’s profitability and can benefit the world beef supply, because Nelore cattle have huge importance in the global beef market [4].

Age at first calving (AFC) is usually utilized in breeding programs as an indicator trait of female sexual precocity, because this information can be easily obtained; the use of this information is directly related to shortening generation intervals and increasing genetic gains [5, 6]. However, selection cannot easily affect AFC because it is a sex-limited trait, is measured after maturity, and its heritability range from low to moderate (0.09 to 0.28) [7–11]. For these type of traits, genome-wide information might allow improving genetic gains through genomic selection [12]. In addition, genome-wide information could be used in association studies (GWAS) to identify candidate regions associated with AFC, and improve our understanding of the genetic basis of sexual precocity in indicine cattle. This knowledge could then be used to improve the accuracy of genomic predictions by using more informative markers and discarding those that generate noise during the predictions.

The use of individual single nucleotide polymorphisms (SNPs) to perform GWAS have some limitations because of the small effect of single markers, which usually do not reach stringent significance thresholds, and also because of the incomplete linkage disequilibrium (LD) between the SNPs and causal variants [13, 14]. On the contrary, the use of a block of SNPs (haplotypes) may provide more robust association analysis, because it improves the resolution of associations and facilitates the approximation of associated markers and possible causal mutations by increasing the LD [15–17].

The aim of this study was to scan for genomic regions associated with AFC in a Nelore cattle population using haplotype allele information, in order to highlight the QTL/genes and genetic mechanisms underlying this trait.

## 3. Material and methods

### 3.1. Ethical statement

The present study was exempt of the local ethical committee evaluation as genomic DNA was extracted from stored hair and semen samples of animals from commercial herds.

### 3.2. Samples

The genotypes used in the current study were provided by the *Zebu Genome Consortium* (ZGC). The full data consisted of 2,273 Nelore samples (995 males and 1,278 females) of different Brazilian herds, born between 1968 and 2008. All samples were genotyped with Illumina BovineHD (~777,000 SNPs).

The association analyses were carried out by considering deregressed estimated breeding values (dEBV) for AFC as response variables, according to Garrick et al. [18].

Prior to deregression, the estimated breeding values (EBV) was obtained for the dataset by considering both the calving and the noncalving (open heifers) females. Open heifers had predicted records that were obtained by adding a penalty of two months to the maximum value of AFC in their respective contemporary groups. Consideration of open heifers was aimed to avoid bias in the estimation of genetic parameters and EBVs [19, 20]. This procedure is based on the assumption that open heifers could have calved if they were bred for longer breeding seasons [20, 21]. The contemporary groups were defined by the concatenation of herd code, year and season of birth, management group identifications (from birth to weaning and from weaning to yearling), and farm (birth, weaning and yearling).

Variance components and EBV were obtained using the DMU software [22]. In the analysis of AFC, a single-trait animal model was fitted, including the fixed effect of contemporary groups and the age of dam as covariates (linear and quadratic effects), as well as a random direct genetic effect and a random residual term. For each animal, the reliabilities of the EBVs were computed based on the corresponding estimates of prediction error variance.

Only genotyped animals that had dEBV with associated reliabilities greater than 25% were kept for further analysis (S1 Dataset), such, that a set of 1,189 animals was considered for association analyses (S2 Dataset). The dEBV average for this samples was equal to -2 (ranging from -46.7 to 60.6) days and reliabilities equal to 0.59 (ranging from 0.25 to 0.94).

### 3.3. Quality control

Genomic data were subjected to quality control (QC) measures, including the maintenance of autosomal markers with minor allele frequencies (MAF) >2%, Hardy-Weinberg equilibrium (HWE) significance at P > 10^−5^ based on Fisher’s exact test, and an SNP call rate > 95%. The following filters were used for sample exclusion: identity by state (IBS) analysis > 95% and the samples in which less than 90% of the genotypes were determined were discarded. The QC was conducted using the GenABEL package, version 1.8–0 [23] of the R software (version 3.2.1) [24].

### 3.4. Genotype phasing

Haplotype assembly requires the identification of the chromosome (maternal or paternal) in which a certain allele is located. Therefore, the haplotype phase was determined using the SHAPEIT software version 2.r837 [25], considering all genotyped animals (N = 2,273).

### 3.5. Linkage disequilibrium

LD between markers was analyzed to determine the number of SNPs per haplotype block, because high-LD markers minimize the occurrence of recombination within the blocks [26]. Therefore, LD was estimated as the squared correlation of allelic frequencies (r^2^), following Hill and Robertson [27]:

$$r^{2}={\frac{\left(freq.AB*freq.ab-freq.Ab*freq.aB\right)}{\left(freq.A*freq.a*freq.B*freq.b\right)}}^{2}$$ (1)

where, freq.A, freq.a, freq.B and freq.b denote the frequencies of A, a, B, and b alleles, respectively; and freq.AB, freq.ab, freq.Ab and freq.aB denote the frequencies of the haplotypes AB, ab, Ab and aB in the population, respectively.

LD between markers was performed using blocks of 5, 10, and 15 markers with a sliding window for each SNP. The window size that resulted in stronger LD was chosen to perform the association study. LD was analyzed using the Plink software, version 1.9 [28, 29].

### 3.6. Haplotypes

With the phased haplotypes of autosomal chromosomes, the haplotypes were constructed with blocks size chosen based on high r² between the SNPs in the LD analysis and sliding window of one marker. The R package GHap v1.2.1 [30] was used to build the haplotype blocks (HapBlock) and identify alleles (HapAllele). Therefore, genotypes were scored as 2, 1, or 0, corresponding with the presence of two copies, one copy, or the absence of the HapAllele, respectively. The genomic position in the association analysis was based on the average distance between the first and last SNPs in a HapBlock. HapAlleles with allele frequency lower than 3% were excluded.

### 3.7. Association analysis

Since dEBV reliability varies across individuals, weighted analyses are required to account for heterogeneous variances. The weights were obtained as proposed by Garrick et al. [18]:

$$w=\left(1-h^{2}\right)/\left[\left(c+\frac{1-r^{2}}{r^{2}}\right)*h^{2}\right]$$ (2)

where h^2^ is the heritability of the trait (0.092) estimated using the complete database of AFC recording, r^2^ is the dEBV reliability, and c is a constant that can assume values from 0 to 1. We assumed that c was equal to 0.5.

Statistical analyses were performed using the *ghap*.*lmm* and *ghap*.*assoc* functions of the GHap package. First, the variance was estimated using the maximum-likelihood method and the following mixed linear model:

y=Xb+Zu+e (3)

where *y* is the vector of dEBV, *X* is the incidence matrix relating elements in y to fixed effects *b* (intercept), *Z* is the incidence matrix for random effects *u* (animal), and *u* is a vector of random effects ~ *N* (0, *Kσ*_*u*_^2^), where *K* is HapAllele relationship matrix, and *e* is a random residual vector (with variance-covariance *Wσ*_*e*_^2^), assuming independence of the residuals.

The estimated residuals from the model (3) were considered as adjusted observations for covariance and polygenic effects, these values were adjusted via Genomic Control [31] and were used in the least squares regression analysis to test the association between each haplotype allele and the phenotype.

### 3.8. Analysis of genomic regions

A genome-wide threshold level at 5 × 10^−5^ was selected for this study, as used by Lander and Kruglyak [32]. For Hapalleles with distances less than 1 Mb or those that overlapped, only the one with the lower P value was chosen. The 1 Mb sequence windows that flanked the significant regions of the top five haplotypes were explored using the bovine genome assembly of UMD version 3.1.

The BioMart tool (Ensembl release 89) [33] and the Cattle QTLdb (release 32) [34] were used to search for candidate gene and cataloged QTL, respectively, surrounding the regions of significant haplotype alleles. The results associated with the examined genes were revised using available scientific literature; the Mouse Genome Informatics (MGI) [35], String v.10.5 [36], and GeneCards: The Human Gene Database [37] provided information regarding the contribution and involvement of orthologous genes associated with AFC.

Due to the large number of genes in the region of these HapAlleles and a involvement of some of them in various metabolic pathways, we have discussed only those that have been previously described in association with reproductive traits, as well as performed by Xu et al. [38].

## 4. Results

After filtering through quality control analyses, 511,375 SNPs and 1,189 individuals remained in the analysis. LD analysis indicated that the average of r^2^ between adjacent markers for the blocks of 5, 10, and 15 SNPs (S1 Fig) were 0.37, 0.32, and 0.30, respectively. Thus, the block sizes of 5 SNPs were chosen to perform association studies.

The haplotyping procedure generated 507,384 HapBlocks and 2,238,795 HapAlleles with allele frequency higher than 3%. The mean number of alleles per block ranged from 4.37 (BTA 1) to 1.19 (BTA 25). The distribution of the number of haplotype blocks per chromosome is shown in S1 Fig.

**Fig 1:**
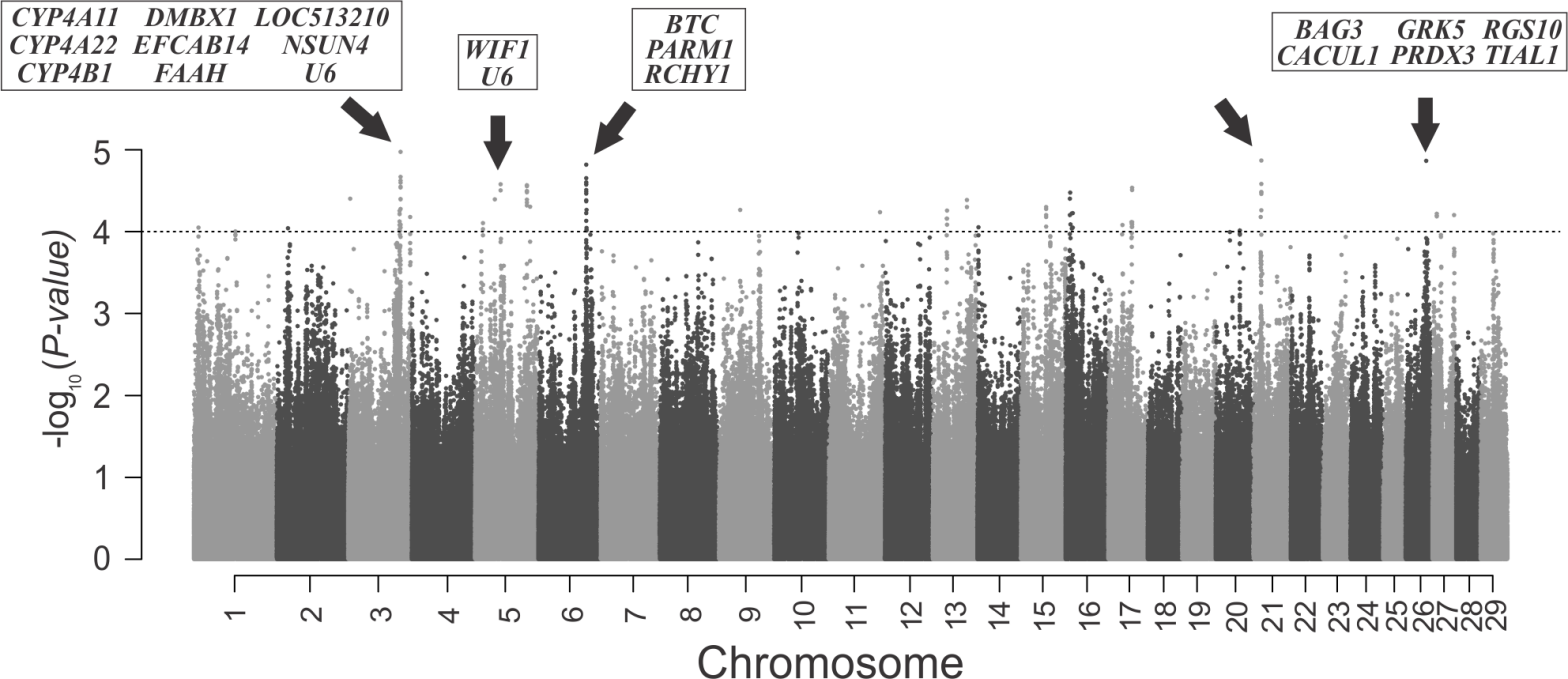
Manhattan plot of the genomic-wide association analysis of the –log_10_ (p-value) of haplotype alleles and AFC trait. Each point in the graph represents a haplotype allele, arrows indicates de top five significant genomic regions and respective genes. The dashed line represent–log10(5 x 10^−5^) threshold.

The results of GWAS between each Hapalleles and AFC in terms of – log10 (P value) are shown in Fig 1, where 68 haplotype alleles with P value < 5 × 10^−5^ were obtained (more details included in S1 Table). The five most significant regions selected for genomic exploration were in the detected peaks of BTA3 (BTA for *Bos taurus* chromosome), BTA5, BTA6, BTA21 and BTA26 (Table 1).

**Table 1:** Top five regions of the GWAS with haplotypic blocks, indicating the chromosomal localization (Chr), start and end positions, the respective SNPs, size and p-values.

BTA	Start (bp)	End (bp)	SNP start	SNP end	Size (bp)	*P-value*
3	99,936,110	99,959,169	rs133273360	rs133299434	23,059	1.07 x10^-5^
5	49,183,430	49,201,226	rs136746086	rs136437853	17,796	2.65 x10^-5^
6	91,649,597	91,663,333	rs134333101	rs135909163	13,736	1.53x10^-5^
21	15,189,159	15,193,691	rs42466473	rs42465819	4,532	1.36 x10^-5^
26	39,765,858	39,773,044	rs110416149	rs41648222	7,186	1.37 x10^-5^

In total, 90 QTL (S2 Table) and 65 candidate genes (S3 Table) were present in these top five regions. Among these, 19 genes were related to previously described reproductive traits (Table 2). Some of the genes that were identified were associated with embryonic implantation in the uterus, development of reproductive organs in females and regulation of progesterone secretion.

**Table 2:** Summary of the genes present in 1-MB windows centered on the haplotypes that were top five significant haplotype alleles.

Gene	Ensembl ID	Position (BTA:start–end [bp])	Description
*U6*	ENSBTAG00000043170 ENSBTAG00000042928	3:99,562,453–99,562,559 5:48,996,775–48,996,881	U6 spliceosomal RNA
*CYP4A11*	ENSBTAG00000037890	3:99,806,666–99,820,784	Cytochrome P450, family 4, subfamily A, polypeptide 22
*CYP4A22*	ENSBTAG00000013481	3:99,918,428–99,933,505	Cytochrome P450, family 4, subfamily A, polypeptide 22
*CYP4B1*	ENSBTAG00000011976	3:99,937,185–99,957,408	Cytochrome P450 family 4 subfamily B member 1
*EFCAB14*	ENSBTAG00000000097	3:100,020,902–100,054,984	EF-hand calcium binding domain 14
*DMBX1*	ENSBTAG00000009625	3:100,217,682–100,223,303	Diencephalon/mesencephalon homeobox 1
*LOC513210*	ENSBTAG00000037858	3:100,285,863–100,300,972	
*FAAH*	ENSBTAG00000007507	3:100,317,761–100,338,715	Fatty acid amide hydrolase
*NSUN4*	ENSBTAG00000015891	3:100,351,395–100,374,287	NOP2/Sun RNA methyltransferase family member 4
*WIF1*	ENSBTAG00000014758	5:48,917,722–49,009,466	WNT inhibitory factor 1
*BTC*	ENSBTAG00000004237	6:91,430,305–91,480,129	Betacellulin
*PARM1*	ENSBTAG00000015919	6:91,597,122–91,723,390	Prostate androgen-regulated mucin-like protein 1
*RCHY1*	ENSBTAG00000007189	6:92,061,508–92,076,962	Ring finger and CHY zinc finger domain containing 1
*CACUL1*	ENSBTAG00000022808	26:39,292,197–39,356,979	CDK2 associated cullin domain 1
*PRDX3*	ENSBTAG00000008731	26:39,672,044–39,681,392	Peroxiredoxin 3
*GRK5*	ENSBTAG00000007981	26:39,702,550–39,930,993	G protein-coupled receptor kinase 5
*RGS10*	ENSBTAG00000002647	26:39,976,325–40,017,296	Regulator of G protein signaling 10
*TIAL1*	ENSBTAG00000004080	26:40,041,404–40,060,834	TIA1 cytotoxic granule associated RNA binding protein like 1
*BAG3*	ENSBTAG00000013641	26:40,118,556–40,141,989	BCL2 associated athanogene 3

Within the top five regions, we found several QTL that were related to reproductive traits (Table 3), and considering the QTL with AFC linkage, we can highlight the age at puberty, daughter pregnancy rate, and conception rate.

**Table 3:** QTL related to reproductive traits that were located in 1-Mb windows near haplotypes.

QTL description	Position (BTA:start–end [bp])	QTLdb ID	PubMed ID
Calving ease	3:84,669,155–102,513,835	15173	21183059
Calving index	3:84,669,155–102,513,835	15172	21183059
Stillbirth	3:84,669,155–102,513,835	15174	21183059
Stillbirth	3:99,853,456–101,636,723	30488	22888914
Calving ease (maternal)	5:49,553,247–51,015,255	106488	27328805
Calving ease	5:48,080,259–48,993,294	24565	24906442
Age at puberty	5:49,485,934–49,485,974	29989	22100599
Interval to first estrus after calving	5:49,399,050–49,399,090	30162	22100599
Stillbirth	5: 49,350,626–51,029,985	30495	22888914
Calving to conception interval	6:87,658,297–92,845,663	126853	28109604
Interval to first estrus after calving	6:87,658,297–92,845,663	126854	28109604
Conception rate	6:91,677,962–91,678,002	57143	23759029
Daughter pregnancy rate	6:91,677,962–91,678,002	57130	23759029
Early embryonic survival	6:91,677,962–91,678,002	57089	23904513
Calving ease (maternal)	26: 39,288,039–39,288,079	52924	21831322
Daughter pregnancy rate	26:39,288,039–39,288,079	52925	21831322
Stillbirth (maternal)	26:39,288,039–39,288,079	52926	21831322
Calving ease	26:39,288,039–39,288,079	52942	21831322
Stillbirth	26: 39,288,039–39,288,079	52944	21831322
Calving ease	26: 39,601,498–41,709,612	15227	21183059

## 5. Discussion

Most of the studies involving haplotypes generated based on the sliding windows have been applied to map alleles associated with human diseases [39]. The use of haplotypes instead of single markers gives putative advantages, the effects of individual SNPs being too small to overcome the stringent significance threshold [14].

The size of the haplotypes might interfere with the success of the analysis, because long blocks lead to the inclusion of non-informative SNPs and increase in the number of rare alleles, whereas short blocks might ignore the informative markers and reduce the power of the association analysis [40, 41]. The use of blocks with 5 SNPs was shown to be appropriate, with regards to the size and average of LD between the adjacent markers. Moreover, the detection of significant signals in the regions of BTA3, BTA5, BTA6 and BTA26, where known candidate genes and QTL of reproductive traits are located, reinforces the suitability of this block size.

The *BAG3*, *DMBX1*, and *PRDX3* genes were annotated in the MGI database [35] with biological function related to abnormalities in fat deposition. Knockout of the *BAG3* genes (cell death suppressor interaction Bcl-2 (Bis)) and *DMBX1* (transcription factor) gave rise to mice with low fat deposition and severe thinness [42, 43]. The *PRDX3* gene is highly expressed in the adipocytes and the suppression or knockout of this gene resulted in obese phenotype in rats and humans [44]. These genes can be pointed out by GWAS because of the positive or negative genetic correlation between body weight and reproductive traits, such as AFC and heifer pregnancies [45], in addition to the proximity of *BAG3* (830 kb) and *PRDX3* (383 kb) with the QTL affecting daughter pregnancy rate. Nelore heifers with early fat deposition can have decreased growth rate, with the nutritional resources directed for reproduction, thus presenting better reproductive performance than that of the heifers of greater weight.

The *eIF* gene family acts at several stages of translation during protein synthesis. Although there were no reports of association between *eIFs* and reproductive traits, functional search in String v10.5 identified the interaction of this family with the genes *TIAL1*, *CACUL1*, and *NSUN4*. The *TIAL1* gene (also known as *TIAR*) encodes protein member of the RNA binding family, acting on post-transcriptional regulation. In rodents, changes in the expression of *TIAL1* severely affects the development of primordial germ cells of gametes (cells that differentiate into sperm and oocytes), being orthologous in cattle [46]. *CACUL1* (or *CAC1*) acts on the activation of the *CDK2* gene involved in cell proliferation processes [47]. In the experiments using mice, inactivation of *CDK2* gave rise to sterile animals, thereby proving to be essential for the development of germ cells in males and females [48]. It is worth mentioning that these two genes are close to the QTL associated with daughter pregnancy rate in BTA26, wherein *TIAL1* is located at 753 kb and *CACUL1* is located at only 4kb, corroborating the idea that these genes have roles in AFC variation. In this context, the *NSUN4* gene has been reported to be differentially expressed in mouse oocytes. *NSUN4* is a candidate gene involved in the association with cumulus cells, responsible for oocyte viability and embryonic developmental competence [38], which could possibly explain their association with AFC in the current study.

Further interaction between the *LOC513210* and *FAAH* genes was found. *LOC513210* belongs to the olfactory gene family that was associated with precocity in Nelore cattle in a previous study[49]. The physiological explanation is that these genes with olfactory function also act on germ cells [49, 50]. The expression of the *FAAH* gene occurs in the uterine epithelial cells and in the myometrium during the estrous cycle in rats. Adjustments in the expression of the *FAAH* gene are carried out by sex hormones and determine the success of uterine receptivity for the implantation of the embryo [51]. In heifers, sheep, and women, the decrease in *FAAH* gene expression has been associated with abortions [52–54]. In cows, the inactivation of *FAAH* can lead to infertility [54].

The *CYP4A22* gene was found to be associated with precocity in Brahman heifers in previous studies [55, 56]. *CYP4A22* belongs to the cytochrome P450 family and is involved in fat metabolism. Nguyen et al. [56] have suggested that this gene acts in response to the progesterone hormone. This hypothesis explains the involvement of *CYP4A22* (having function in the pituitary gland) in the mechanisms of ovarian feedback established with during puberty. Using String, it was possible to detect a relationship between *CYP4A22* and *CYP4A11*, which was the last one associated with perinatal modifications in target tissues that is necessary for the progression of pregnancy [57].

In addition to *CYP4A22*, the *PARM1* gene has also been previously reported to be associated with precocity in heifers [58, 59]. *PARM1* is expressed in the ovaries and encodes proteins involved in cell proliferation the, is regulated by androgens [60]. The *PARM1* gene was also associated with daughter pregnancy rate of Holstein sires [61], and is located within two QTL that are essential for AFC, conception rate, and daughter pregnancy rate. This gene also participates in the regulation of luteinizing hormone and controls progesterone levels in rats [62, 63].

The genes *RGS10*, *WIF1* and *CYP4B1* were reported to be essential for the embryo implantation process in the uterus and for pregnancy viability [64–67]. *RGS10*, located 688 kb close to the daughter pregnancy rate QTL, had its expression identified in the pig endometrium. *RGS10*, which is orthologous in cattle and pigs, can lead to changes in the endometrium during the estrous cycle that can lead to successful embryo implantation [65]. *WIF1* is located 476 kb away from the age at puberty QTL. This gene encodes the regulatory protein of canonical WNT molecules, involved in cell proliferation, differentiation, and migration [66, 68]. Expression studies in the endometrial tissue of heifers suggested the activity of the *WIF1* gene as a molecule moderator, important for the implantation and development of the embryo [66, 67].

The *BTC* gene, localized on the BTA6, 197 kb away from the QTL affecting conception rate and daughter pregnancy rate, is essential for oocyte maturation and development, and fertilization [69]. *BTC* acts as the mediator of luteinizing hormone effects [70], which regulates functions related to puberty, such as oocyte maturation and ovulation [71].

Gurgan et al. [72], aiming to identify markers capable of predicting the quality of human oocytes detected the presence of the *RCHY1* gene as a possible hormone regulator involved in follicular development, further emphasizing that this gene is close (383 kb) to the conception rate and daughter pregnancy rate QTL.

In a transcriptomic study using granulosa cells from the ovarian follicles of heifers, the *GRK5* gene was detected in association with the maturation of granulosa cells [73]. These cells are important for the hormonal regulation occurring in the gonads and for viability of the female gamete [73, 74]. It is noteworthy that this gene is found 414 kb of daughter pregnancy rate QTL.

The *EFCAB14* gene encodes a calcium ion binding protein and belongs to the EF-hand calcium binding gene family. There are still no reports available in the literature on the biological pathways in which genes from this domain (domain 14) are involved. However, this gene family has been associated with reproductive traits in women and cattle [75, 76].

Another candidate gene, located in the BTA3 and BTA5, encodes the U6 spliceosomal RNA, and had paralogous genes associated with AFC in a different population of Nellore cattle, as reported in a previous study [77]. In BTA5, this gene is close (498 kb) to an important QTL related to AFC, which is the age at puberty QTL.

There were no known genes or QTL related to the reproductive traits among the eight QTL and four genes annotated in the vicinities of the significant haplotypes of BTA21 (S2 and S3 Tables). This may be due either to the lack of knowledge regarding the functions of these genes or the existence of non-annotated genes in this region. It is noteworthy that the present study detected association in regions different from that reported by previous studies on the Nelore breed [77–79]. This can be because of herd particularities, such as the extent of LD, allelic frequencies, sample size, and statistical approaches [79], in addition to the use of haplotypes in the current study.

The number of significant peaks dispersed across the genome (Fig 1) confirms the polygenic nature of the trait AFC. Therefore, the current study was able to reveal genomic regions putatively associated with the reproductive performance of cattle.

The proximity of the QTL with the association regions and the involvement of QTL in reproductive traits (e.g., calving ease, daughter pregnancy rate, calving rate, embryonic survival, stillbirth, and conception rate) support the results obtained for the examined genes. Moreover, our results support the involvement of the identified regions in AFC.

## 6. Conclusion

The use of haplotypes allowed the detection of chromosomal regions associated with AFC. In these regions, genes and QTL related to reproduction were found. These results provide the basis for further studies that aim to elucidate the mechanisms underlying the roles of the examined genes during AFC expression.

Thus, a better understanding of this mechanism will allow the use of specific genotypes as a guide in animal genetics improvement programs, and will enable the construction of cheaper, low-density panels for the evaluation of specific genotypes that are advantageous to selection.

## Supporting information

S1 Fig Number of haplotype blocks per chromosome. (TIF)

S2 Fig Plot for average of r^2^ to adjacent markers for blocks of 5, 10, and 15 SNPs. (TIF)

S1 Table Haplotypes alleles higher the threshold. (CSV)

S2 Table List of QTL related to age at first calving. (CSV)

S3 Table List of candidate genes related to age at first calving. (CSV)

S1 Dataset Fenotype information of animals. (RAR)

S2 Dataset Genotype information of animals. (GZ)
