# Supplementary figures and images for: Genome-wide association study using haplotype alleles for the evaluation of reproductive traits in Nelore cattle
Source: PLoS One. 2018 Aug 8;13(8):e0201876. doi: 10.1371/journal.pone.0201876 (PMC6082543; doi:10.1371/journal.pone.0201876)

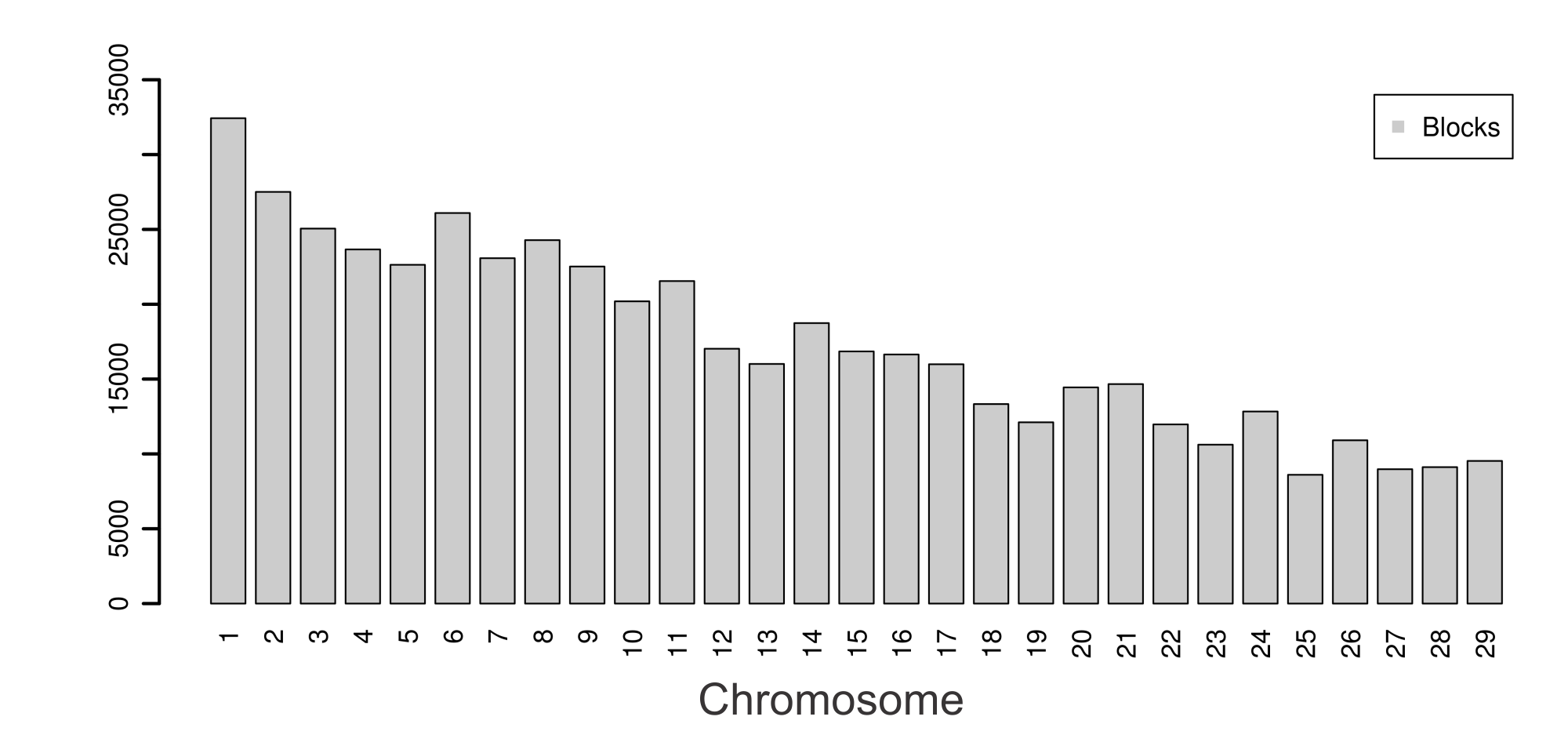

Supplement: S1 Fig — (TIF) [file pone.0201876.s001.tif]

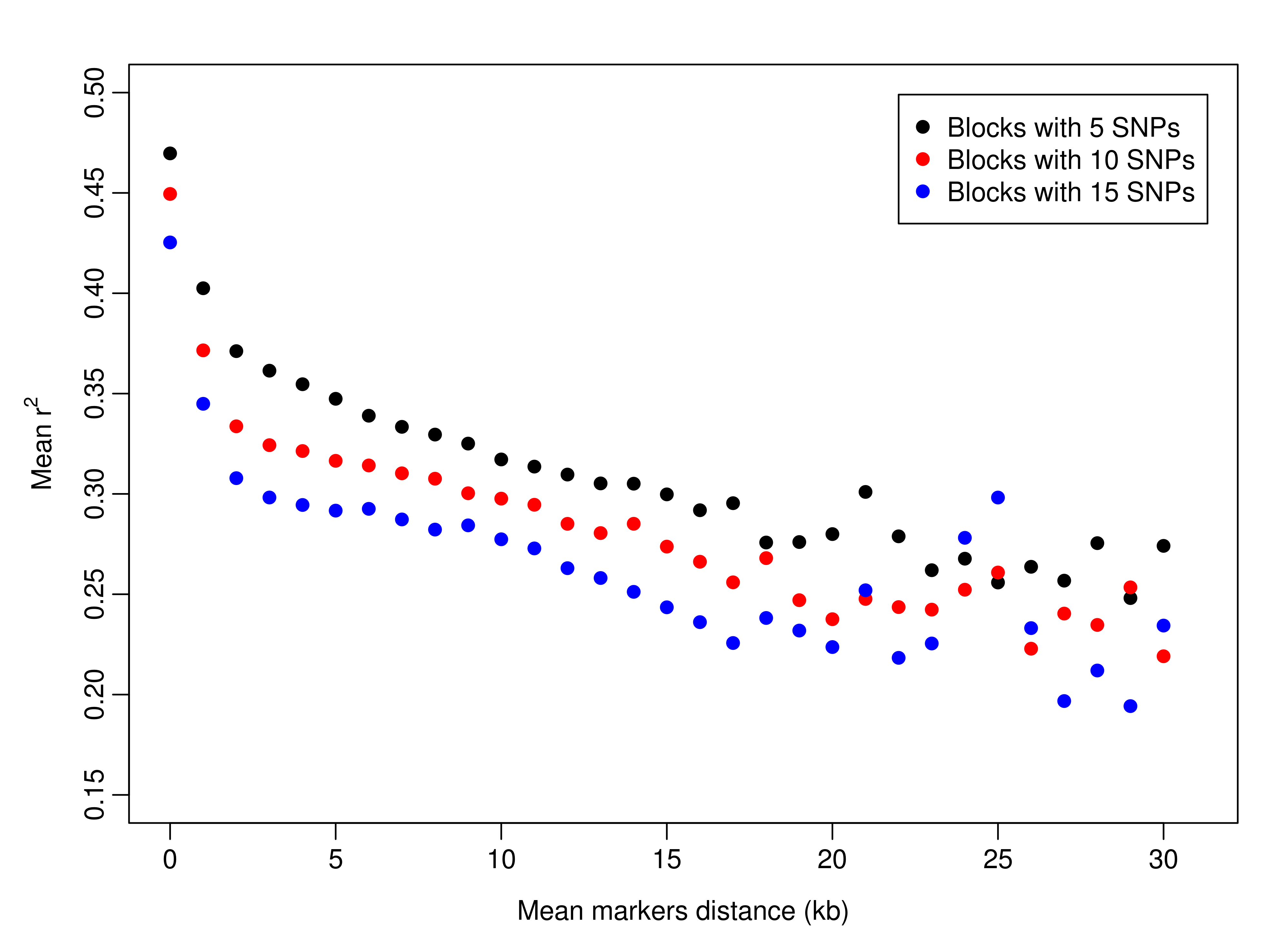

Supplement: S2 Fig — (TIF) [file pone.0201876.s002.tif]
